# The Presence of Yin-Yang Effects in the Migration Pattern of Staurosporine-Treated Single versus Collective Breast Carcinoma Cells

**DOI:** 10.3390/ijms222111961

**Published:** 2021-11-04

**Authors:** Frank A. H. Meyer, Dominik Kraus, Alexander Glassmann, Nadine Veit, Jochen Winter, Rainer Probstmeier

**Affiliations:** 1 Neuro- and Tumor Cell Biology Group, Department of Nuclear Medicine, University Hospital, Medical Faculty, University of Bonn, Venusberg-Campus 1, 53127 Bonn, Germany; frankymeyer@arcor.de (F.A.H.M.); nadine.veit@ukbonn.de (N.V.); r.probstmeier@gmx.net (R.P.); 2 Department of Prosthodontics, Preclinical Education, and Material Sciences, University Hospital, Medical Faculty, University of Bonn, Welschnonnenstr. 17, 53111 Bonn, Germany; dominik.kraus@ukbonn.de; 3 Life Science Inkubator, Ludwig-Erhard-Allee 2, 53175 Bonn, Germany; alexander.glassmann@h-brs.de; 4 Department of Immunology and Cell Biology, University of Applied Science Bonn-Rhein-Sieg, Campus Rheinbach, von-Liebig-Str. 20, 53359 Rheinbach, Germany; 5 Oral Cell Biology Group, Department of Periodontology, Operative and Preventive Dentistry, University Hospital, Medical Faculty, University of Bonn, Welschnonnenstr. 17, 53111 Bonn, Germany

**Keywords:** cell migration, breast carcinoma, invasion, staurosporine, yin-yang effect

## Abstract

Background: Staurosporine-dependent single and collective cell migration patterns of breast carcinoma cells MDA-MB-231, MCF-7, and SK-BR-3 were analysed to characterise the presence of drug-dependent migration promoting and inhibiting yin-yang effects. Methods: Migration patterns of various breast cancer cells after staurosporine treatment were investigated using Western blot, cell toxicity assays, single and collective cell migration assays, and video time-lapse. Statistical analyses were performed with Kruskal–Wallis and Fligner–Killeen tests. Results: Application of staurosporine induced the migration of single MCF-7 cells but inhibited collective cell migration. With the exception of low-density SK-BR-3 cells, staurosporine induced the generation of immobile flattened giant cells. Video time-lapse analysis revealed that within the borderline of cell collectives, staurosporine reduced the velocity of individual MDA-MB-231 and SK-BR-3, but not of MCF-7 cells. In individual MCF-7 cells, mainly the directionality of migration became disturbed, which led to an increased migration rate parallel to the borderline, and hereby to an inhibition of the migration of the cell collective as a total. Moreover, the application of staurosporine led to a transient activation of ERK1/2 in all cell lines. Conclusion: Dependent on the context (single versus collective cells), a drug may induce opposite effects in the same cell line.

## 1. Introduction

The aggressiveness of many tumour cells correlates with their potential to migrate and, consequently, to evade from the primary tumour, which, further on, may enhance the formation of metastasis. Epithelial to mesenchymal transition (EMT) has often been stated as the preferential explanation for the conversion of tumour cells into a migratory phenotype, i.e., independent of additional genetic mutations, harbouring migratory and invasive properties [1]. The relevance of EMT has mainly been regarded in the context of single migrating tumour cells, as collective cell migration, the second major type of tumour cell migration, seems to be relatively independent of features typical for EMT [1]. Nevertheless, collective tumour cells can change their molecular and cellular properties with respect to their single counterparts and, thereby, may increase their migratory potential [2]. In this context, we have previously demonstrated that collective migration of thyroid tumour cells enables them to overcome unfavourable substrate areas [3].

Recently, it has been suggested that the different types or modes of migration, i.e., single or collective, should not be seen as distinct entities present in a discrete on or off manner, but instead—due to the integration of different signals raised, for example, by cell-to-cell or cell-to-extracellular matrix (ECM) contacts—as continuous types of conversions [4]. Assuming such a model, the consequences of one and the same extracellular signal on the migration behaviour could show considerable variations, depending on whether tumour cells are present as single entities or within a cell collective.

Besides the already mentioned cell signalling events raised by cell-to-cell and cell-to-ECM contacts, growth factor and cytokine signalling also contribute to the behaviour of tumour cells, including their migration patterns [5]. Such phenomena can be investigated in different experimental systems that may vary in their level of complexity. The small kinase inhibitor staurosporine (SSP) is an alkaloid derived from the bacterium *Streptomyces staurosporeus*. Amongst other small kinase inhibitors, SSP has relatively quickly lost clinical interest, as it exhibited a too-broad inhibition profile based on the fact that its molecular structure overlaps with the adenosine portion of ATP [6]. However, more recently, new delivery technologies led to a kind of comeback of SSP in the context of cancer treatment [7]. In particular, application of SSP nanoparticles led to an almost complete growth inhibition of multidrug-resistant breast tumours in animal models [8]. In a detailed study performed by Karaman et al. [9], SSP has been shown to interact with KD values of less than 3 μM, with 253 out of 290 kinases tested, the latter one representing around 55% of the predicted human kinome. On the cellular level, SSP inter-alia interferes with cell migration, proliferation, differentiation, and survival in a multifaceted manner [10,11]. We recently have shown that SSP mediates the conversion of small-cell lung carcinoma (SCLC) cells into a neuron-like process-bearing phenotype [12], whereby the broad pattern of SSP-induced effects is more restricted, with different SSP analogs that exhibit higher substrate specificity [13]. In addition, SSP can also induce the reversible formation of resting giant cells, as demonstrated by us for A549 non-SCLC cells [14].

Breast carcinoma, the leading cancer entity of women in most developed countries, comprises many different biological entities. The 5-year survival rate strongly depends on the absence or presence of a metastatic stage (99% versus 27%) [15]. As metastasis formation in general strongly correlates with cell migratory events during early stages of cancer formation, a detailed elucidation of cell migration processes in breast cancer should help to increase treatment options [16]. Classical breast cancer classification distinguishes between luminal A and B, HER2-overexpressing, and basal (triple-negative) tumours [17]. To gain more insight into the migratory potential and flexibility of breast carcinoma cells, here, we have used three breast cancer cell lines that differ in their oestrogen receptor (ER), progesterone receptor (PR), and epidermal growth factor receptor 2 (HER2) expression pattern, as well as in their metastatic potential: Luminal-like MCF-7 breast cancer cells (ER-, PR-positive, HER2-negative) harbour properties of a differentiated mammary epithelium and express epithelial markers such as E-cadherin, β-catenin, or cytokeratin 18, but are negative for the mesenchymal markers vimentin and smooth-muscle actin. These cells possess only a low migratory potential in vitro and do not induce metastasis in mice [18]. The cell lines MDA-MB-231 (ER-, PR-, HER2-negative) and SK-BR-3 (ER-, PR-negative, HER2-positive) are mesenchymal-like, highly invasive, and metastatic [19,20,21], although the highly metastatic potential of SK-BR-3 cells has been questioned [22]. These three cell lines are originally derived from metastatic sites and well-characterised with respect to their pathological abnormalities in their expression pattern of potential therapeutic genes [20,23]. They belong to the ten most cited cell lines in PubMed [23], indicating the presence of considerable datasets that allow the integration of our results into desired frameworks. However, it has to be mentioned that the categorisation of breast carcinoma cell lines is still controversial [24].

In the present study, single or collective breast carcinoma cells were treated with SSP on different substrata. Dependent on the cell line and the starting conditions, such treatments revealed a multifaceted reaction pattern. Our data highlight a multifaceted drug-response of tumour cells in the context of cell–cell and cell–extracellular matrix interactions that, in its extreme, can lead to an inverse response. Thus, the impact of a drug should be investigated in parallel for different cellular parameters (such as cell proliferation, migration, differentiation, or cell death) in order to exclude the presence of opposite yin-yang effects [25].

## 2. Results

**Figure 1:**
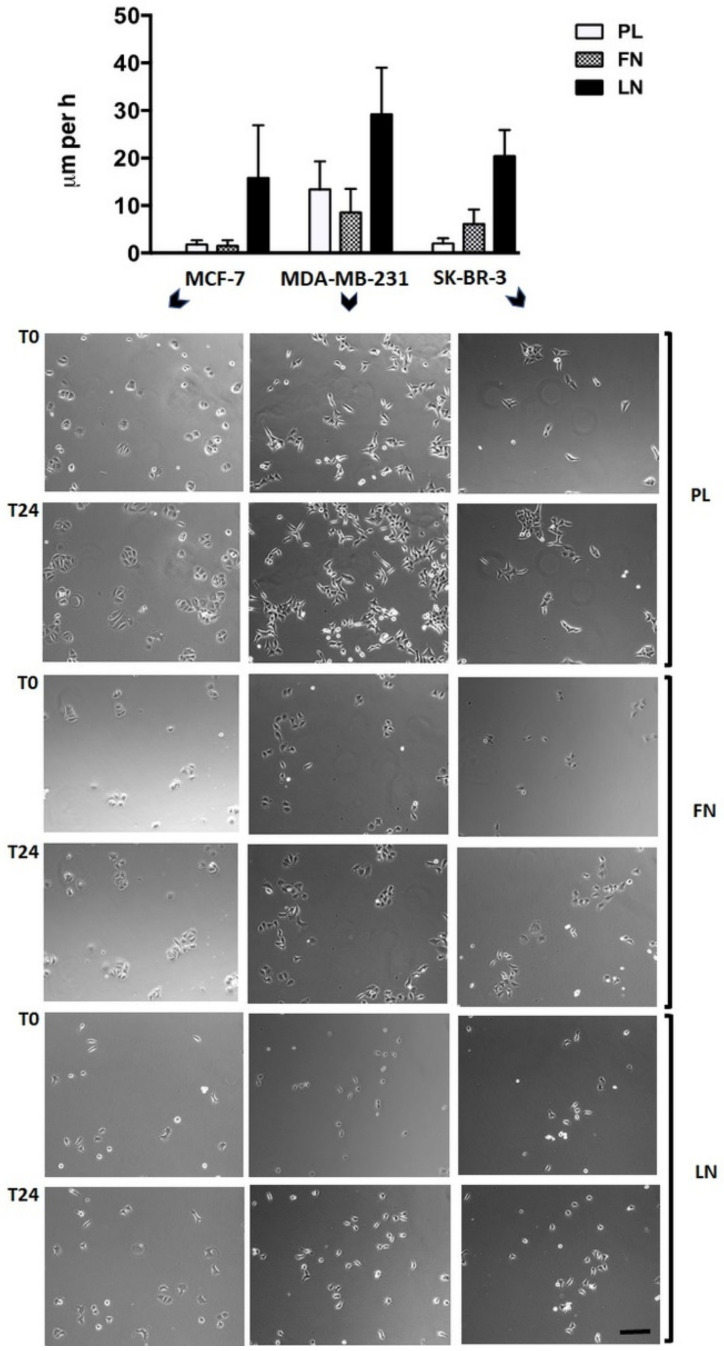
Single-cell migration of breast carcinoma cells on plastic (PL), fibronectin (FN), or laminin (LN) surfaces as revealed by video time-lapse analysis. Histogram shows the velocity of breast carcinoma cells that were analysed for 24 h (given in µm per h + SD). Tracing of the migratory paths was accomplished with the software “Image J” and “CellTracker”. Per cell line and substratum, at least 20 cells were analysed. Selected micrographs show breast carcinoma cells that had been cultivated for 24 h on a plastic (PL), a fibronectin (FN), or a laminin (LN) substratum. Micrographs of identical sections at the beginning (**T0**) and after 24 h (**T24**) of the culture period are shown (bar, 50 μm). Notice that in some combinations, such as MCF-7 or SK-BR-3 cells cultivated on PL, almost identical positions of immobile but proliferating cells are present, whereas considerable but variable cell movements occur in other combinations, such as MCF-7 or MDA-MB-231 cells cultivated on LN (also reflected by the large SD values in the histogram).

Single human breast carcinoma cells exhibit a complex migration pattern on different substrata. Breast carcinoma cell lines MCF-7 (MCF), MDA-MB-231 (MDA), and SK-BR-3 (SKB) showed a diverse migration potency dependent on the cell line and the substratum, when seeded at low density on cell culture plastic (PL), fibronectin (FN), or laminin (LN) surfaces and analysed by video time-lapse for 24 h (Figure 1). In general, individual cells exhibit a variable velocity within the cell population analysed, a fact that is reflected by the high SD of the mean values. In more detail, MCF cells were almost immobile on PL (1.0 ± 0.9 μm/h) and FN (0.8 ± 1.2 μm/h), but mobile on LN (15.8 ± 11.1 μm/h). MDA cells were mobile on all three substrata, i.e., 13.4 ± 5.9 μm/h on PL, 8.5 ± 5.0 μm/h on FN, and 29.2 ± 9.8 μm/h on LN. SKB cells were weakly mobile on PL (2.0 ± 1.1 μm/h), but mobile on FN (6.1 ± 3.1 μm/h) and LN (20.4 ± 5.5 μm/h). Cells that were more mobile often harboured processes or expressed an elongated shape. Thus, common breast carcinoma cell lines possess a variable migration capacity on different substrata. 

**Figure 2:**
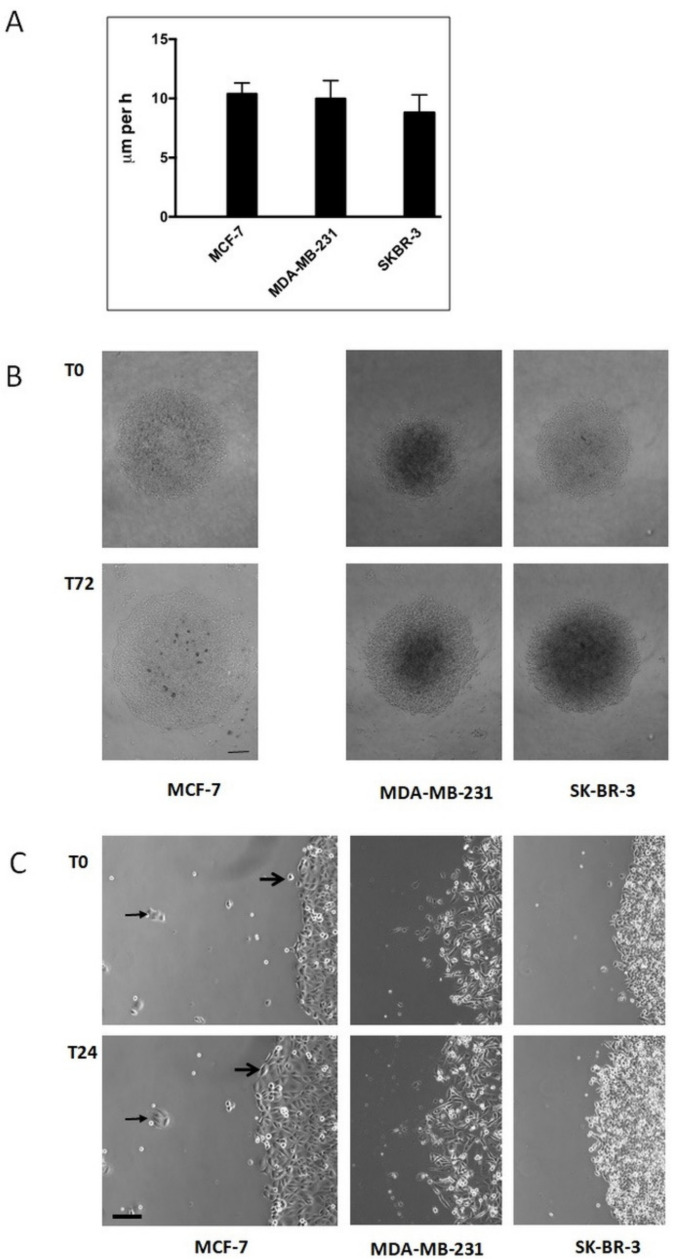
Collective cell migration of breast carcinoma cells cultivated on plastic surfaces. (**A**) Histogram shows the increase in the diameter (given in μm per h + SD) of circular areas covered with a confluent layer of breast carcinoma cells after three days in culture (for MCF-7 cells, see subfigure (**B**)). At least twelve circular areas were measured per experiment and at least three independent experiments per cell line were performed. (**B**) Low-magnification micrographs of representative circular areas covered with a confluent layer of breast carcinoma cells at the onset of the experiment (T0) and three days later (T72) (bar, 200 μm). (**C**) High-magnification micrographs of the borderline of circular areas covered with confluent layers of breast carcinoma cells at the beginning of the experiment and one day later (T24) (bar, 60 μm). For MCF-7 cells, big arrows mark the changing position of a single cell that becomes integrated in the cell collective. Small arrows mark the constant position of a small cell cluster outside the cell collective.

Immobile single human breast carcinoma cells gain a migratory potency when present in a collective. We next analysed, in endpoint studies, breast carcinoma cells that were allowed to migrate as a collective on a PL surface (Figure 2). Under these conditions, all three cell lines were able to migrate. If we assume that individual cells migrate on a straight line, we found a velocity of 10.4 ± 0.9 μm/h for MCF, of 10.0 ± 1.5 μm/h for MDA, and of 8.8 ± 1.5 μm/h for SKB cells (Figure 2A). These data demonstrate that MCF and SKB cells retain a migratory potency when present in a collective on otherwise unfavourable substrata for single-cell migration, such as cell culture plastic, a phenomenon we have recently shown to be present in thyroid carcinoma cells [3]. Moreover, video time-lapse analysis of MCF cells revealed that otherwise immobile single cells became mobile when integrated into a cell collective (Figure 2B, right panel, small arrows), and that small clusters of MCF-7 cells were immobile during the time period analysed (Figure 2B, right panel, large arrows). These data point to the fact that, at least for MCF cells, the size of a collective has to exceed a critical cell number, before migration takes place.

**Figure 3:**
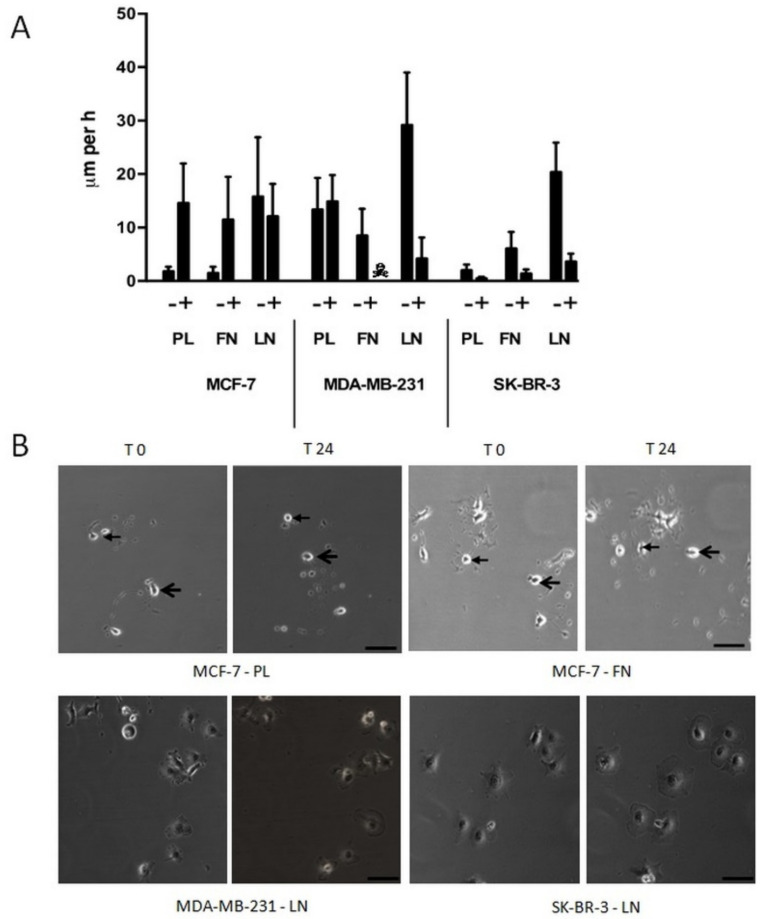
Single-cell migration of breast carcinoma cells on plastic (PL), fibronectin (FN), or laminin (LN) surfaces in the absence or presence of 50 nM of SSP, as revealed by video time-lapse analysis. (**A**) Histogram shows the velocity of breast carcinoma cells that were analysed for 24 h (given in μm per h + SD). With the exception of MCF-7 cells on LN and MDA-MB-231 cells on PL, the differences between untreated and SSP-treated cells are statistically significant, as determined by Student’s *t*-test. ☠: MDA-MB-231 cells on FN did not survive SSP treatment. (**B**) Selected micrographs of breast carcinoma cells that had been cultivated for 24 h in the presence of 50 nM of SSP on a PL, FN, or LN substratum. Micrographs of identical sections at the onset of the experiment (T0) and 24 h later (T24) are shown. Notice the changed positions of cells of MCF-7 cells cultivated on PL or FN. In each case, the position of two cells is indicated by small or large arrows (bar, 40 μm). Notice the presence of immobile flattened MDA-MB-231 and SK-BR-3 cells cultivated on LN.

Immobile single human breast carcinoma cells show complex changes in their behaviour and morphology when cultivated in the presence of SSP. We have recently shown that SSP induces dramatic changes in cell morphology, such as the induction of neurite-like processes [12] or the formation of polyploid giant cells [14]. Therefore, we decided to cultivate single breast carcinoma cells in the presence of 50 nM of SSP (Figure 3), the highest concentration that was still non-toxic (as tested with the LDH assay, see the Materials and Methods Section) for all three cell lines when cultivated on PL. Under these conditions, MCF cells cultivated on PL or FN became mobile (PL: 14.6 ± 7.4 μm/h; FN: 11.5 ± 8.0 μm/h), whereas the velocity on LN was slightly decreased in comparison to untreated cells (15.8 ± 11.1 μm/h versus 12.1 ± 6.1 μm/h). The migratory potency of MDA cells on PL was not influenced by SSP (13.4 ± 5.9 μm/h versus 14.9 ± 4.9 μm/h). Unexpectedly, MDA cells cultivated on FN did not survive in the presence of SSP. When cultivated on LN, MDA cells started to flatten during the 24 h incubation period, whereby cell velocity was considerably reduced (29.2 ± 9.8 μm/h versus 3.9 ± 3.4 μm/h). The appearance of such cells resembled that of immobile giant cells that we have already observed for SSP-treated A549 cells [14]. SKB cells cultivated on PL remained weakly immobile in the presence of SSP (2.0 ± 1.1 μm/h) but showed extensive cell flattening that was also observed for cells cultivated on FN or LN, whereby cell movement was strongly inhibited (FN: 1.4 ± 0.8 μm/h; LN: 3.3 ± 2.0 μm/h). 

**Figure 4:**
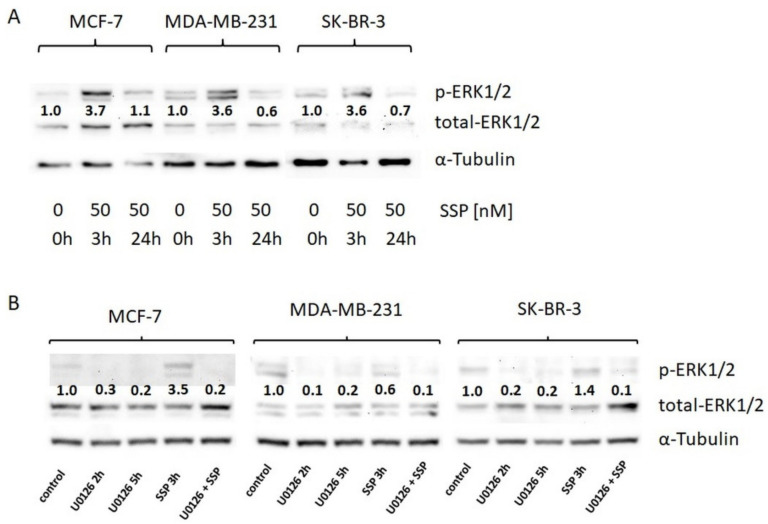
Western blot analysis of ERK1/2 activation in breast carcinoma cells. (**A**) Breast carcinoma cells were cultured in DMEM, 10% FCS, and directly solubilised (0 h) or solubilised after incubation with 50 nM of SSP for the indicated time spans. (**B**) Breast carcinoma cells were directly solubilised (control) or, before solubilisation, treated for the indicated time spans with the MEK inhibitor U0126 (20 μM) or for 3 h with 50 nM of SSP either in the absence or presence of 20 μM of U0126. α-Tubulin was used as a loading control. Numbers show fold change compared to controls (set as “1.0”).

The exposure of single breast carcinoma cells to SSP is accompanied by a transient activation of the canonical MAPK/ERK1/2 pathway, which could be abolished in the presence of the MEK inhibitor U0126 (Figure 4). This activation seems to be independent of the outcome at the cellular level, i.e., induction of migration or appearance of immobile giant cells, suggesting a diversification in more downstream activation/signalling events.

**Figure 5:**
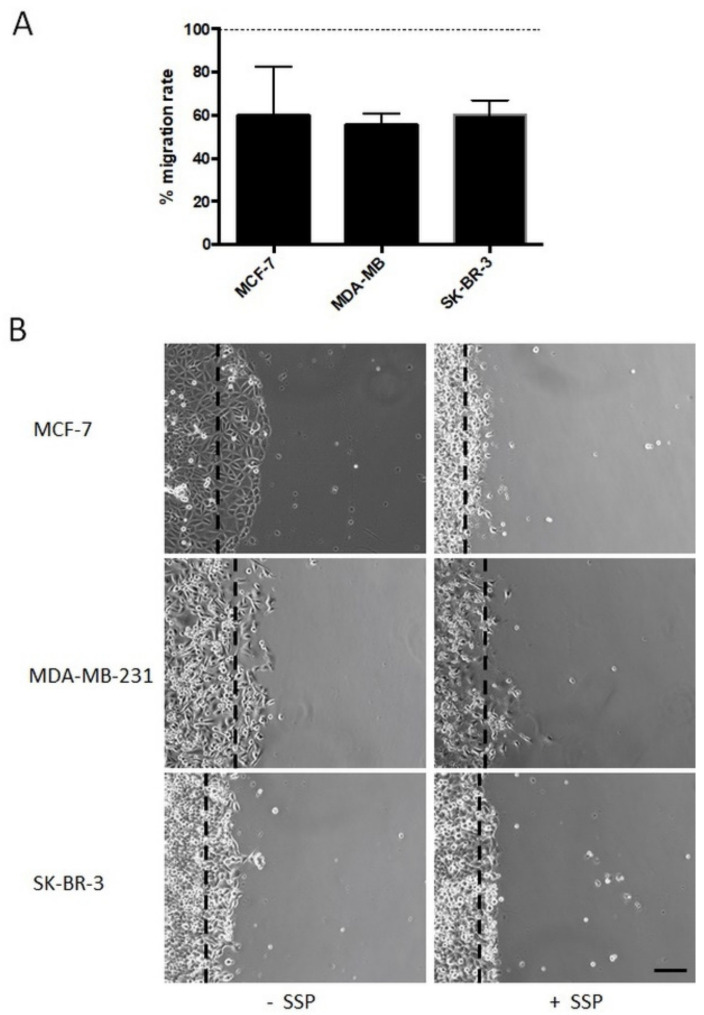
Collective cell migration of breast carcinoma cells cultivated on plastic surfaces in the absence or presence of 50 nM of SSP. (**A**) Histogram shows the relative SSP-provoked inhibition of the migration rate (given in %), i.e., the change in the diameter of a circular area covered with a confluent layer of breast carcinoma cells after three days of culture (for MCF cells, see subfigure (**B**)). Per experiment, at least twelve circular areas were measured and at least three independent experiments per cell line were performed. The dashed line represents the migration rate of untreated cells that was artificially set as 100%. (**B**) Micrographs show the borderline of a circular area covered with a confluent layer of breast carcinoma cells after a cultivation period of 24 h in the absence or presence of 50 nM of SSP (bar, 60 μm). Dashed lines mark the position of the border at the beginning of the experiment.

Bulk migration of collective human breast carcinoma cells is inhibited in the presence of SSP. We then analysed breast carcinoma cells that were allowed to migrate as a collective in the presence of 50 nM of SSP on a PL surface (Figure 5). In endpoint studies, the overall migration was reduced in the range of 40% for all cell lines (Figure 5A), a result which is in partial contrast to the data revealed for single cells, in particular for SSP-treated MCF cells (see Figure 3). As shown in Figure 5B, the borderline of collective MCF cells became clearly altered in the presence of SSP, whereby cells became smaller, exhibited reduced cell-to-cell interactions, and produced processes. Moreover, occasionally but reproducibly, individual cells left the collective and migrated as single entities.

SSP-mediated inhibition of bulk migration is provoked by inhibition of cell velocity and reduced directed migration. To obtain a more detailed inspection, video time-lapse analyses were performed. The recorded data were analysed for two main parameters: (1) cell velocity and (2) migration directionality. Whereas the first parameter can be simply calculated, for the second one, we had to establish specific scripts generated with the help of the statistical program “R”. Such R-scripts allow: (1) to embed the randomly located coordinates of individual cells within the surface of a cell culture dish at the beginning of the experiment (T0) into the intercept (0, 0) of a 2D coordinate system, and (2) the performance of statistical tests as well as graphical plots and, thus, the calculation of the angle spectrum within which a defined part of the whole cell population migrates (enabled by the fact that the migration of all cells can be located at the same starting point in an artificial coordinate system; see also the Materials and Methods Section). This angle would be zero degrees if all cells migrate in the main direction, i.e., the *x*-axis, and 180 degrees if the cells migrate perpendicular, i.e., along the *y*-axis. Thus, the degree of directional migration can be calculated. 

When we first calculated the mean velocity of individual border cells after 24 h, we obtained for untreated MCF cells mean values of 2.4 ± 3.1 μm/h, for MDA cells 6.0 ± 7.5 μm/h, and for SKB cells 2.5 ± 3.4 μm/h. In the presence of SSP, the velocity was increased by 29% to 3.1 ± 3.5 μm/h for MCF cells, and decreased by 43% to 3.4 ± 5.0 μm/h for MDA cells and by 44% to 1.4 ± 2.3 μm/h for SKB cells. These values are considerably lower in comparison to the data obtained in endpoint studies after three days of culture (see Figure 2A), which hints at the presence of higher velocities after longer time periods. This interpretation is supported by video time-lapse analyses for such time periods. Unfortunately, based on technical reasons, long-time experiments could not be undertaken in a sufficient number.

**Figure 6:**
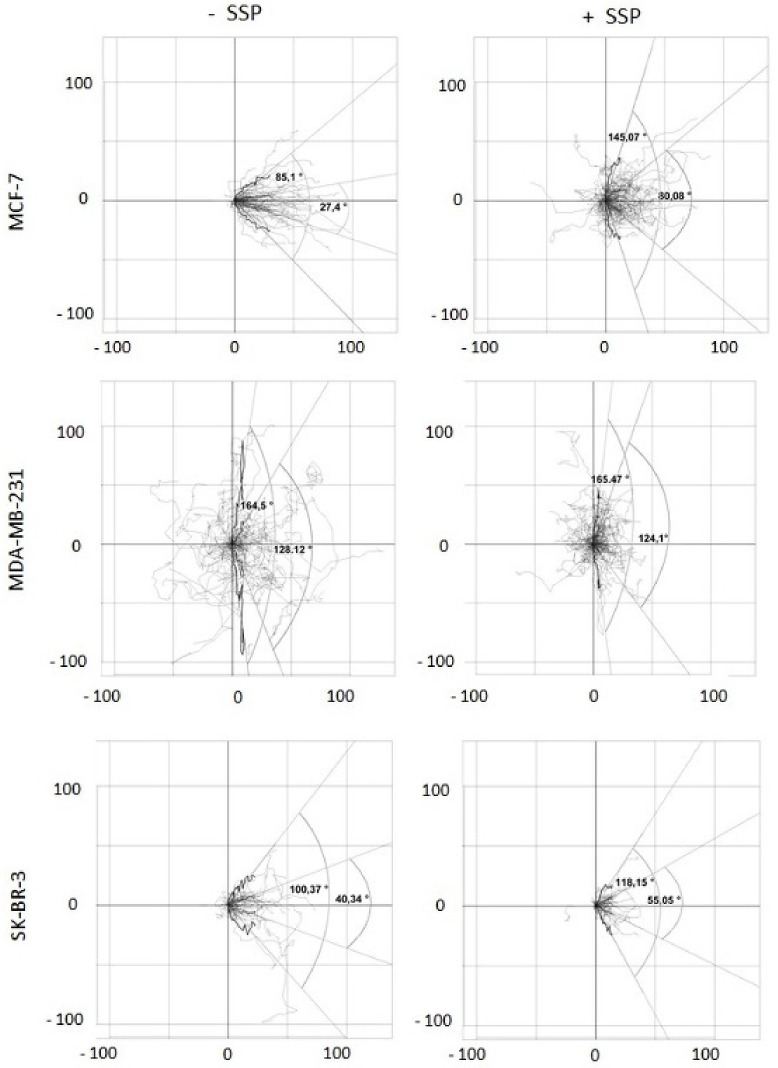
Two-dimensional analysis of the migration pattern of collective border breast carcinoma cells. Collective breast carcinoma cells were allowed to migrate for 24 h in the absence (-SSP) or presence (+SSP) of 50 nM of SSP. The paths of at least 40 carcinoma cells derived from two independent experiments were recorded and integrated into a 2D coordinate as a series of coordinates. With the help of especially designed R-scripts, the different starting points of all cells at T0 were superimposed in the intercept of the “zero” lines in all subfigures, and then the corresponding paths (shown in light grey) were integrated into the 2D coordinate system. Thereby, the paths were reoriented such that the main direction of migration on the abscissa was oriented to the right (see Figure 5B as a comparison). Each black curved line represents a “summarised path” which was calculated for each time point for the position of all individual cells analysed at a certain time point (total time span 24 h, divided from T0 to T72 in 20 min intervals). The individual coordinates of the “summarised path” are based on box and whisker plots for each time point. Hereby, on the X-coordinate, the medians of all 20 min intervals for all cells are presented, whereas on the Y-coordinate, the corresponding lower and upper whisker values or the lower Q25 and upper Q75 quartile values are provided. This set of individual coordinates represented by the summarised paths allows the generation of regression lines. The raise of such regression lines can vary between 0 and 90 degrees, or 0 and –90 degrees, respectively. The angles which can thereby be generated express borders defined by either the lower and upper whiskers (wider angles) and encompass the majority of all path segments, or the lower Q25 and upper Q75 quartile (narrower angles) and encompass 50% of all path segments. Numbers at the X- and Y-axes represent μm.

When migrating collective breast carcinoma cells were examined after 24 h, according to this scheme, it turned out that in SSP-treated MCF as well as in SKB cells, but not in MDA cells, the portion of the paths that cells migrated in the y-dimension increased, reflected by the presence of wider angles (Figure 6). Based on a box plot analysis, the angle defined by the lower and upper whisker (based on the y-coordinates) was considerably increased in SSP-treated MCF cells, from 85.12 to 145.07 degrees, remained constant in MDA cells (164.50 versus 165.47 degrees), and was slightly increased in SK-BR-3 cells (100.37 versus 118.15 degrees). Comparable values were obtained for the narrower Q25 to Q75 quartile angle: for MCF cells, 27.40 versus 80.08 degrees, for MDA cells, 128.12 versus 124.10 degrees, and for SKB cells, 40.34 versus 55.05 degrees.

**Figure 7:**
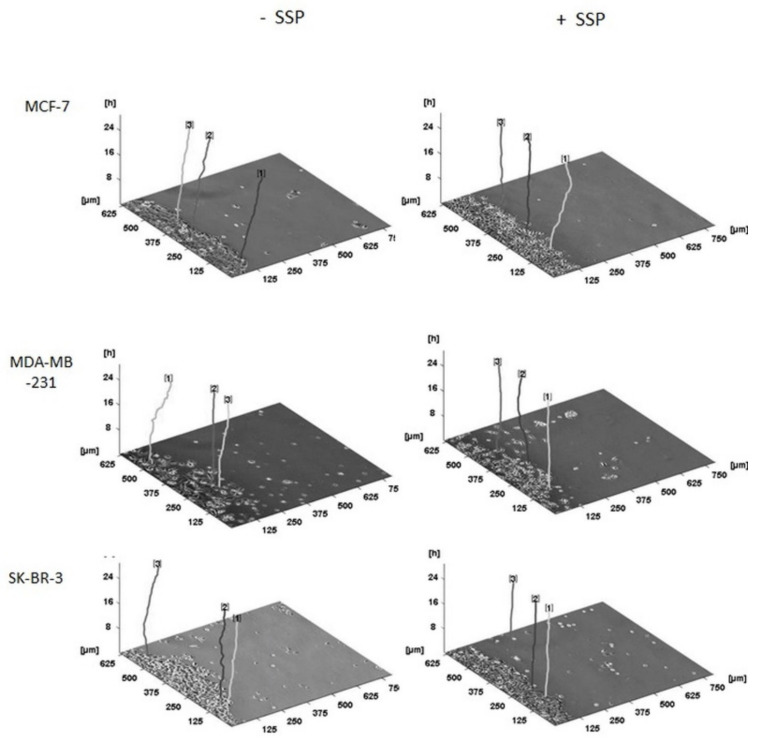
Three-dimensional migration pattern of selected collective borderline breast carcinoma cells. Collective breast carcinoma cells were allowed to migrate for 24 h in the absence (–SSP) or presence (+SSP) of 50 nM of SSP. The main direction of migration is oriented to the right. The time-dependent (*z*-axis) paths of three representative carcinoma cells (located in the borderline of the cell collective) per cell line and treatment are shown. For all paths, the endpoint on the *z*-axis is located at the 24 h position. Thus, the total length of the individual lines may differ, based on a variation of the curvature of the paths. Processing of the primary data was performed with the program “CellTracker” that allows the documentation only in pixel format. One pixel was then converted to 1.25 μm. Numbers at the top of the paths were automatically set by the program.

A three-dimensional presentation of the migration pattern of individual collective cells as shown in Figure 7 documents the “raw data” used for Figure 6, whereby the given representative individual cells are located at their original and, thus, different positions inside the borderline of the cell collective. 

**Figure 8:**
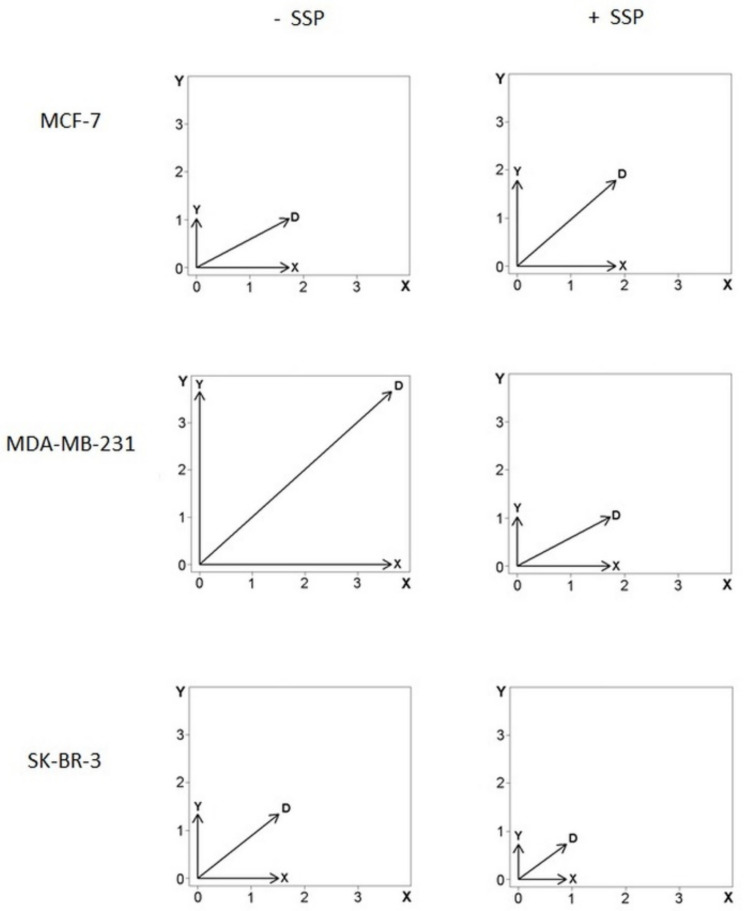
Vector diagrams of cell velocities from individual collective border breast carcinoma cells. Data were summarised from cells that were analysed as shown in Figure 6. The *X*-axis is oriented to the main direction and represents the changes of the mean velocity given by the x-coordinates (μm per h) oriented into the main direction of the overall migration of the collective (see text for details and Figure 5 for orientation). The *Y*-axis represents the changes of the mean velocity given by the y-coordinates (μm per h). The diagonal, D, represents the sum of the vectors X and Y with the vector’s magnitude defined by ||D|| = (sqrt(X^2^ + Y^2^)) in μm per h. For details, see text.

As the data shown in Figure 6 do not allow a discrimination of proportional changes present in the X- as well as in the *Y*-axis, as it is the case for MDA cells, we have also summarised the data in the form of vector diagrams (Figure 8). 

The horizontal (X) and vertical (Y) arrows represent the mean velocity values for each dimension (see the Materials and Methods Section for details) for all cells analysed. The diagonal arrow, D, represents the vector’s sum, with its magnitude defined by ||D|| = sqrt(X^2^ + Y^2^). In comparison to the control situation, in SSP-treated MCF cells, the length of D is elevated by 27%, whereby the X-value remains almost constant (+6%), whereas the Y-value is increased by 75%, i.e., the SSP-induced inhibition of migration in the main X-direction is mainly due to an increased migration rate in the Y-direction, but not to a decreased cell velocity. Consequently, the X to Y ratio is decreased from 1.7 to 1.0. In SSP-treated MDA as well as SKB cells, the length of the D, X, and Y values is considerably decreased, i.e., the SSP-induced inhibition of migration in the main X-direction is mainly due to a decreased velocity, but not to an altered direction of individual migrating cells. Thus, the X to Y ratio also remains almost unchanged. 

## 3. Discussion

We have described here the migration pattern of single and collective breast carcinoma cells in the absence or presence of SSP. Our main findings were (for a quantitative summary, also see Table 1):

(i) Breast carcinoma cells can gain a migratory potential on otherwise unfavourable substrates, when present within a collective (MCF and SKB cells on a PL substratum), a phenomenon we have already shown to occur in thyroid carcinoma cells [3]. 

(ii) SSP-dependent promotion of single-cell migration is cell-line dependent. Whereas SSP promotes migration of MCF cells on PL and FN but does not alter the migration pattern on LN, it clearly inhibits the potent migration of MDA and SKB cells on a LN substratum via the induction of a giant cell polyploid phenotype. Such a phenotype in general seems to possess a restricted migration potency [14].

(iii) SSP induces migration in single but inhibits overall migration in collectives of MCF cells via a perturbation of the directional migration in individual cells.

Recently, we have provided evidence that drugs inhibiting the MEK/ERK1/2 module can simultaneously induce the activation of one cellular response (migration) and the suppression of another (proliferation) in one and the same cell line (i.e., Cal-62 thyroid carcinoma cells), a phenomenon that we have designated as the yin-yang effect [25]. In the present study, we showed that in MCF cells, a specific drug can provoke another kind of yin-yang effect characterised by the induction or suppression of a comparable response (single versus collective cell migration) in one and the same cell line. 

Zambrano and co-workers [26] have recently shown that SSP suppresses cell viability of Her2/neu-positive breast cancer cells (human JMT-1 cells, SMF (MMTV-neu) murine-derived mammary tumour cells) in the lower nM range. In our study, Her2/neu-positive SK-BR3 cells (see http://www.merckmillipore.com/DE/de/product/fordocumentation, accessed on 30 July 2021) tolerated SSP concentrations in the higher nM range, at least for several days without significant signs of cell death, indicating a higher variability in SSP-sensitivity in different Her2/neu-positive human breast cancer cells.

In general, a yin-yang effect can be defined as an impact of a physical and/or molecular nature that leads to opposite outcomes, such as potentiation or inhibition, of molecular or cellular parameters in a defined biological system, such as well-characterised primary cells or cell lines. Yin-yang effects of repressive versus repressive or adhesive versus repulsive nature have been described during neural development and disease [27,28]. With respect to cancer biology, yin-yang effects have been described inter-alia for the action of signalling molecules and transcription factors [29]. In 1991, a transcription factor ubiquitously expressed in mammalian cells was named yin-yang 1, according to its context-dependent activation or repression of transcription [30]. Additionally, in cancers, yin-yang 1 exhibits the opposite function, thereby activating or repressing tumour cell proliferation and growth [30]. Irritatingly, the term “yin-yang” is not consistently used in the literature. With respect to cancer biology, it is also used to distinguish between genes or pathways that promote (yin type) or those that repress cancer, or at least promote a normal cellular state (yang type) [31]. Moreover, in the context of tumour therapy, desired tumour-targeting effects of therapy have been described as yin effects, whereas counteracting reactive host responses have been designated as yang effects [32]. 

The verification of a yin-yang effect that is present within different contexts of cell migration was only possible via the introduction of specific R-scripts into analysis software, as described above. To our knowledge, a video time-lapse-based quantitative analysis concerning the directionality of collective cell migration on the single-cell level has not been performed yet. Especially for MCF cells, it became thereby evident that although the velocity of individual collective border cells was slightly increased by 29% in the presence of SSP (2.4 ± 3.1 μm/h versus 3.0 ± 3.5 μm/h), the overall migration of the complete collective (as indicated by the size of a big collective cell cluster, see Figure 2 and Figure 5) was considerably decreased. We believe that such scripts could be helpful for comparable questions, where the directed migration patterns of individual cells within a collective need to be quantified. 

Although the migration pattern of individual SSP-treated MCF cells within a collective is less oriented in comparison to their untreated counterparts, it is not completely random. Indeed, if a random migration scenario of completely independent cells would be the case, an area of initially collective cells would “thin out” in the presence of SSP, which is initiated by the random emigration of individual cells. As this is predominantly not the case, SSP treatment only partially reduces but not completely abolishes intercellular communication processes. As already described in the Results Section, it is important to recognise that rarely but reproducibly, individual cells leave the collective, a phenomenon that has to be seen with respect to invasion and metastasis formation. Thus, although the cell collective as a whole is inhibited in its net migration, some individual cells are able to conquer tumour-free areas. It is likely that collective cell migration is present at a higher level of complexity than that of individual cells and exhibits supracellular features [33]. Hereby, the degree of supra-cellularity can vary [33], whereby phenomena as described by us have, to our knowledge, so far not been documented.

At present, the analysis of yin-yang effects in the context of cell migration has not received special adequate attention in the scientific literature. Opposite effects of one and the same intrinsic protein or drug have been described for hepatocellular carcinoma cell lines. Whereas in one cell line, overexpression of the cellular senescence-inhibited gene (CSIG) promoted cell migration and ERK-activation, in another cell line, opposite effects were detected [34]. We have described somehow similar effects in a thyroid carcinoma cell line, where inhibition of MEK/ERK1/2 signalling inhibits proliferation, but promotes migration, an effect that can be prevented by a parallel inhibition of the PI3/Akt pathway [26]. Thus, our study is the first example that describes the relevance of yin-yang effects in context-dependent (single versus collective) tumour cell migration.

In breast cancer, collective cell migration seems to represent the predominant invasion mode, whereby the composition of intercellular contacts varies between invasive ductal carcinoma (adherens junctions) and invasive lobular carcinoma (CD44-mediated) [35]. These authors have also provided evidence that multicellular groups in primary breast cancer tissue retain epithelial characteristics without a complete EMT conversion, whereby cell individualisation is higher in ILC than in IDC [35].

At a first glance, the impact of a drug is predominantly dependent on the cell type or cell line: SSP can induce process formation (in SCLC cells), a polyploid giant phenotype (A549-, MDA-, or SKB cells), or a migratory phenotype (MCF cells). At a second glance, the impact of a drug in one and the same cell line can be considerably modified via cell–cell and cell–extracellular matrix interactions. Thus, it seems necessary to catalogue the various effects of a drug not only according the respective cell types or cell lines, but also according to the respective cellular contexts. Only then can a satisfying impact profile be established for drugs.

## 4. Materials and Methods

Cell lines and culture conditions: The human breast carcinoma cell lines MCF-7, MDA-MB-231 (both from DSMZ, Braunschweig, Germany), and SK-BR-3 (CLS, Eppelheim, Germany) were maintained in DMEM, supplemented with 10% foetal calf serum (FCS), 100 U/mL penicillin–streptomycin, and 2 mM L-glutamine [36]. Cell lines were routinely tested for the presence of mycoplasm.

Western blot analysis: Total extracts of breast carcinoma cells from cultures of 70% to 80% confluency (which should mimic the conditions of video time-lapse studies) were lysed for 10 min on ice in RIPA (Radio-Immunoprecipitation Assay) buffer (50 mM Tris/Cl- pH 8.0, 150 mM NaCl, 1% Igepal CA-630, 0.5% sodium desoxycholate, 0.1% SDS) containing protease inhibitors (0.5 mM PMSF, Roche complete Mini ULTRA mix) and phosphatase inhibitors (10 mM sodium fluoride, 1 mM sodium orthovanadate, 10 mM 2-glycerophosphate). The extract was centrifuged at 4 °C for 10 min (15,000× *g*) and the supernatant was used for further analysis, as described in [37]. Antibodies towards phospho-ERK1/2 and total ERK1/2 were from Cell Signalling and antibodies towards β- tubulin were from Santa Cruz.

Cell toxicity assay: Cytotoxicity was analysed with the “LDH Cytotoxicity Assay Kit” from Roche based on the release of cytosolic lactate dehydrogenase (LDH) into the cell culture supernatant by damaged cells. Briefly, 10,000 cells (in 100 μL culture medium) were seeded per well in 96-well plates, and 24 h later, cells were exposed to different concentrations of SSP for 24 h. After treatment, LDH activity was determined in the cell culture supernatants. In parallel, cells that had been treated identically were lysed in order to determine total LDH activity.

Single-cell migration assay: 4000 cells in 500 µL of culture medium were applied to 24-well plates (kept untreated or precoated with bovine fibronectin or murine EHS laminin at a concentration of 20 µg/mL) and allowed to adhere overnight. Two to three hours prior to time-lapse analysis, compounds or DMSO (both diluted in 500 µl of culture medium) were added per well. Video time-lapse microscopy and analysis were performed as described in [38]. Briefly, plates were transferred to a heated (37 °C), gassed (5% CO_2_/air), and humidified chamber fitted onto an inverted microscope (Nikon Ti-E) with a motorised cross-stage. Images were recorded every 20 min for 24 h. Cell movement and densitometry were tracked and analysed with the ImageJ plugin MTrackJ (www.imagescience.org/meijering/software/mtrackj/) and the software Cell Tracker (http://celltracker.website/index.html, accessed on 30 July 2021). The latter was also used as a plugin for MatLab (https://mathworks.com/products/matlab.html, accessed on 30 July 2021).

Collective cell migration assay: Cell suspensions (5000 cells/µL) in a total volume of 3 µL were seeded on defined regions in Petri dishes and allowed to adhere for 2 to 4 h. After floating the dishes with culture medium, the adherent and confluent cells occupied a circular area. The diameters of the areas (12 per dish) were determined microscopically after floating (T0) and a second time after a after a culture period of 72 h (T1), with or without experimental compounds. Video time-lapse analysis of collective cells, micrographs, and analyses were performed as described above for the single-cell migration assay.

Analysis of the migration pattern of breast carcinoma cells: The program “CellTracker” v. 1.1 upgraded with the program “MatLab R2018b” to accelerate the workflow was used to track randomly chosen cells at the borderline of circular confluent cell spots. The cell paths, i.e., series of two-dimensional (2D) coordinates obtained in 20 min intervals, were analysed with modified scripts developed with the statistical program “R”, that was designated here as “R-scripts” (Frank A. H. Meyer et al., manuscript in preparation). Beside the execution of statistical tests, R-scripts allow the separate analysis of split 2D cell path intervals (provided in a vector format) for each dimension. Hereby, the cell path intervals, i.e., vectors, were rotated such that their main direction of cell migration was oriented from left to right inside the *X*-axis. Consequently, a corresponding perpendicular vector was oriented in the *Y*-axis. Moreover, all the vectors were shifted with respect to the intercept of an artificial 2D coordinate system, whereby consequently, the starting point of each cell path at T0 was set at the coordinates (0, 0). At first, it was tested whether individual border cells in treated and untreated cell collectives differ by comparing their medians and other ranks of their migration rate within the x-coordinate (Kruskal–Wallis test). Second, it was tested whether such border cells behave differently with respect to their migration rate within the y-dimension. In case such an effect occurs in treated cells, the variances of their y-coordinates should differ with respect to untreated cells (Fligner–Killeen test). Highly significant differences were present, when H0, i.e., the null hypothesis, could be rejected for *p*-values below 1%. For the graphical presentation of the data, box plots were used as a standard and their main values were transferred into angle spectra (details are outlined in the Results Section and the corresponding figure legends).

## 5. Conclusions

The drug-induced inhibition of collective cell migration can result from two different mechanisms: (i) an inhibition of the migration of single cells independent of their orientation within the collective, and (ii) an inhibition of the directed migration of single cells dependent of their orientation within the collective (with respect to the borderline). Whereas the first mechanism is strictly accompanied by a decrease in the net migration path length of single cells, for the second mechanism, the path length may remain unchanged. Obviously, such opposite yin-yang effects could interfere with the therapeutic efficiency of drugs. Moreover, these effects are context- (single versus collective migration) and cell line-dependent, and thus document the need for individual patient-based treatment strategies.

## Figures and Tables

**Table 1:** Single and collective migration pattern of breast carcinoma cells on PL, FN, or LN substrata in the absence or presence of SSP after 24 h.

	Single-Cell Migration									Collective Cell Migration (Individual Border Cells)	
Substratum	PL			FN			LN			PL	
SSP	no	yes		no	yes		no	yes		no	yes
Cell line											
MCF-7	-		++	-		++	++		+	+	+
MDA-MB-231	++		++	++		☠	++		+/-	++	+
SK-BR-3	+/-		+/-	++		+/-	++		+	+	+/-

-: velocity ≤ 1.5 μm/h; +/-: velocity ≥ 1.5 ≤ 2.0 μm/h; +: velocity ≥ 2.0 μm/h ≤ 5.0 μm/h;

++: velocity ≥ 5.0 μm/h; ☠: cell death.

## Data Availability

The datasets used and/or analysed during the current study are available from the corresponding author upon reasonable request.
